# Physiotherapeutic conduct in amyotrophic lateral sclerosis

**DOI:** 10.1590/S1516-31802006000600011

**Published:** 2006-11-01

**Authors:** Andreza Martinez Pozza, Milene Karenine Delamura, Clarissa Ramirez, Nelson Iguimar Valério, Laís Helena Carvalho Marino, Neuseli Marino Lamari

**Keywords:** Amyotrophic lateral sclerosis, Rehabilitation, Breathing exercises, Neurodegenerative diseases, Quality of life, Esclerose amiotrófica lateral, Reabilitação, Exercícios respiratórios, Doenças neurodegenerativas, Qualidade de vida

## Abstract

Amyotrophic Lateral Sclerosis (ALS) is a fatal progressive neurodegenerative disease with multifactorial etiology for which, so far, there is no effective medicinal treatment. However, by means of kinesiotherapy intervention and patient guidance and care, physiotherapy can delay physical functional losses, muscle fatigue and immobility of the joint-muscle system, thereby improving the quality of life. This survey had the aim of reviewing the physiotherapeutic conduct currently used in ALS cases. Monthly monitoring is recommended, with changes in goals and conduct at each stage of the disease, activities to be pursued around the home, and emphasis on stretching, muscle strengthening, posture adequacy and respiratory kinesiotherapy.

## RESUMO

Esclerose lateral amiotrófica (ELA) é uma doença neurodegenerativa progressiva, fatal, de etiologia multifatorial e sem tratamento medicamentoso efetivo até o momento. Entretanto, a fisioterapia, por meio de intervenções cinesioterapêuticas e orientações aos pacientes e aos cuidadores, ameniza a perda físicofuncional, a fadiga muscular e a imobilidade do sistema músculo-esquelético, melhorando a qualidade de vida. Buscou-se neste trabalho sugerir os procedimentos fisioterapêuticos utilizados atualmente na ELA. Indica-se realizar acompanhamentos mensais com mudanças nos objetivos e nas condutas a cada fase da doença e propõe-se acompanhamento das atividades em domicílio, com ênfase em alongamentos, fortalecimento muscular, adequação postural e cinesioterapia respiratória.

## INTRODUCTION

Amyotrophic lateral sclerosis (ALS) was first described by Charcot in 1869^1^ and is also known as Lou Gehrig's disease, as a tribute to an American baseball player who died of ALS in 1941.^2^ It is a progressive neurodegenerative disease that involves the motor cortex, the brainstem and the motor neurons of the spinal cord.^3^ It is believed that the etiology of ALS is multifactorial and includes genetic and environmental factors. The mechanism for neural cell death caused by genetic factors remains unclear, but neural injury occurs due to an excess of free radicals resulting from mutations of a gene that codes for an enzyme called superoxide dismutase.^4^ Environmental factors and toxic, autoimmune, infectious and metabolic processes may trigger the disease if the patient has a predisposition.^1^

This non-systematic review of the literature was conducted on publications from 1995 to 2005. Even so, it was difficult to find references to physiotherapy in amyotrophic lateral sclerosis (ALS) cases because of the small number of articles. Three important databases, PubMed, Medline and SciELO, were accessed to find texts on this topic. Book chapters were also consulted, specifically to confirm information and to locate additional references.

The evolution of the disease is rapid, with death generally occurring between two and five years after diagnosis. The common signs are weakness and muscle atrophy, cramp, fasciculation, alterations in gait and alterations in reflexes and tonus.^5^ The bulbar symptoms are dysphagia, dysarthria, dysphonia and respiratory alterations. This latter is the main cause of death.^6^

The incidence of the disease is reported to be from 0.4 to 2.4 cases per 100,000 inhabitants, with a prevalence of 2.5 to 7.0 cases per 100,000 inhabitants^7^. In Brazil, the characteristics are similar to those found in Europe and North America, with a higher prevalence in men (gender ratio of 3:2) and an average onset age of 57 years.^8^

The diagnosis is clinical, complemented by laboratory tests and electroneuromyography,^5^ and the El Escorial diagnostic criteria are used, classifying into four levels of diagnostic certainty: suspected, possible, probable and definitive.^9^ The differential diagnosis is obtained by neuroimaging.^10^

There is currently no effective medicinal treatment. Riluzole is the only approved agent for clinical use, and it extends survival by a few months.^11^ Recent studies on the benefits of creatine and vitamin E, which have neuroprotective action that improves residual muscle contractility and neuron function,^12^ have also reported positive results with regard to increased survival and delayed evolution of the disease.^13^

Multidisciplinary intervention has the principal objective of preserving quality of life and functionality.^14^ Physiotherapy offers assistance during the development of the disease, and attempts are made to alter its objectives and conduct in each phase of the disease in order to delay the evolution of the symptoms.^15^

### Stages of the disease

Dal Bello-Hass et al.^7^ classified ALS into stages of functional dependence, with the aims of simplifying the guidelines for clinical attendance, maintaining maximum functional mobility for patients and improving their quality of life. These stages are:

- Stage 1: Preserved functional independence, muscle weakness with changes in resistance. Physiological support and continuity of normal physical activities are recommended.
- Stage 2: Involvement of the distal musculature with the use of orthoses indicated in some cases. The same conduct as in Stage 1 is maintained, but cardiopulmonary and neuromuscular conditioning and physical-functional training are added.
- Stage 3: Moderate functional limitations and more susceptibility to fatigue. Patients require wheelchairs and may use orthoses for the arms.
- Stage 4: This is the start of the severe phase, with changes in the lower limbs. Continuation of the exercises from Stage 2 is necessary, with the addition of exercises aimed at preventing muscle contracture and guidance about positioning when lying down.
- Stage 5: Functional dependence requiring stretching exercises and manual therapy performed to reduce the muscle and joint pains, spasticity and muscle fasciculation. Electrical stimulation and hydrotherapy help with the muscle instability, as well as the use of orthoses.
- Stage 6: This is the maximum state of dependence. Patients are sometimes immobile, with involvement of the respiratory system. Cardiopulmonary physiotherapy is of great importance at this stage, as are changes in posture and a homecare program. The physiotherapeutic techniques of Stages 1 and 2 should be continued.

These authors concluded that this division into stages might help physiotherapists to detect the clinical phase of the disease. It might also give the correct indication of exercises and guidance, in order to delay complications such as muscle contracture, pain and respiratory impairment.^7^

Piemonte and Ramirez^15^ also suggested three classification stages for functional dependence (independent, semi-independent and dependent), together with specific physiotherapeutic conduct for each phase. These authors also recommended daily exercises taught to patients and caregivers in the outpatient clinic. The three stages were described as follows:

- Independent stage: Motor ability is preserved, with the patient walking and performing normal daily activities. There is a slight reduction in muscle strength and susceptibility to fatigue. The main aims are to keep motor functioning stable for as long as possible, to avoid muscle retractions and joint deformities, to reeducate about posture and to give guidance on the use of orthoses.
- Semi-independent stage: Individuals present difficulty in performing daily activities and the use of wheelchairs is necessary. This is the start of respiratory system involvement, with dyspnea during moderate effort. Stretching, muscle strengthening, torso posture exercises and respiratory kinesiotherapy exercises are recommended. These procedures increase flexibility, reduce cramp, strengthen the musculature and improve the posture.
- Dependent stage: Patients require caregivers to assist them in performing day-today activities because of the evolution of the symptomatology. Preservation of joint mobility with emphasis on the pelvic and scapular regions, preservation or improvement in control over the torso and neck, respiratory training and postural changes are recommended.

### Physiotherapeutic interventions

Gómez Fernández and Calzada Sierra^14^ used a rehabilitation program to maintain functional adaptation and prevent complications from muscle immobility. Six patients with ALS underwent seven hours of rehabilitation per day, three times a week, for four weeks, while avoiding fatigue. They used respiratory and motor kinesiotherapy, by means of stretching and muscle strengthening exercises, postural reeducation, facial mimicking exercises and body relaxation. The results were significant: four of the six patients presented with improvement in their quality of life with early intervention.^14^ Piemonte and Ramirez^15^ also concluded that early intervention by a multidisciplinary team offered improved quality of life and increased longevity.

However, Pedroso et al.^16^ did not achieve the same results. They administered daily physiotherapy sessions to ALS patients in their homes over a six-month period, using motor and respiratory exercises to delay disease evolution and reduce the costs for the patients. There were two groups: independent and semi-independent patients. From these groups, a test group was formed composed of five independent and three semi-independent individuals who underwent functional, fatigue and muscle force evaluations. A control group was formed, composed of five independent and three semi-independent patients who did not perform these exercises. After evaluating the patients every three months over a one-year period, no significant differences were seen between the two groups.^16^

With the aim of guiding caregivers and family members regarding patients with ALS, Duran et al.^17^ enrolled 95 patients and their caregivers in a study over a three-year period. The patients were initially evaluated by a multidisciplinary team and then they were reevaluated by physiotherapists at two-month intervals to assess their degree of muscle strength, tonus, deformities, amplitude of movement, pain, edema and daily-life activities. The physiotherapeutic follow-up emphasized the importance of doing tasks around the house, in accordance with the evolution of the disease and the difficulties that had been reported by caregivers and family members. The results were satisfactory, and 80% of the patients were included in the study. Information on how to provide a better quality of life in relation to the disease was supplied to the patients.

### Fatigue and exercises

Drory et al.^18^ discussed patients’ susceptibility to fatigue during physiotherapeutic activities, in relation to the duration of the disease. The authors selected 25 patients: 14 performed moderate daily exercises and 11 did not perform physical activities. These patients were evaluated every three months over a 12-month period, using the manual strength test,^19^ Ashworth Scale of Muscle Spasticity,^20^ functional scale,^21^ fatigue severity scale,^22^ quality-of-life questionnaire^23^ and visual analog pain scale.^24^ The authors observed that, in the first three months, there were fewer losses according to the functional and Ashworth scales, but not using the other parameters. Over the first six months there were no significant differences between the groups. However, there was a tendency towards less deterioration in relation to all the scales for the group that did exercises. From the ninth to the twelfth month, there were insufficient patients in either group to continue the research, but the authors concluded that performing moderate exercises regularly might help in the patients’ day-to-day activities.^18^

Ramirez^25^ studied fatigue in patients with ALS and found that there are some citations of studies on athletes undergoing strenuous exercise who evolved with ALS. There are few studies to prove such data, but it is believed that moderate and regular physical exercise should be recommended for patients with this disease, while respecting their functional difficulties and performing periodic reevaluations to follow up the evolution of the disease, with the possibility of changing the exercises.

Van den Berg-Vos et al^26^ concluded that vigorous physical activity, such as heavy work or sports like athletics, is a risk factor for ALS, after analyzing several famous athletes who were affected by this disease.

Scarmeas et al.^27^ reported that there is a supposed relationship between athletes undergoing strenuous exercising and the development of ALS, although these studies are still inconclusive. These authors analyzed 431 athletes between 1992 and 2000 by means of body mass index calculations, the El Escorial diagnostic criteria and the history of the disease. They also evaluated the characteristics of premorbidity and low body weight. They established a case group (athletes affected by ALS) and a control group (athletes affected by other neurological diseases) and put them through the same types of physical activities during the study period. The authors concluded that intense physical activity could not be correlated with triggering ALS, since there was no progression of the disease in the athletes with ALS (they had only slight reductions in muscle strength in comparison with the control group) or onset of the disease in the control group.

Chio et al.^28^ demonstrated in a large retrospective study that there was a highly significant relationship between professional soccer players’ risk factors and the presence of ALS. The players studied were in the Italian First Division (series A) or Second Division (series B) and the study period was from September 1, 1970, to June 30, 2002. Information on dates of birth, ages, dates when these players entered professional soccer teams, positions they played in and length of time in the profession were collected from club records. Players who presented onset of symptoms of the disease or died up to December 31, 2001, were considered to have been at risk.

There were 7,325 professional soccer players in the study. Eighteen cases of players who developed ALS were identified over the study period. Three of these individuals were excluded because they had not been born in Italy and ten because they had been players since before 1970. Thus, five cases were defined over the 137,078 person-years of this follow-up (defined in the years 1981, 1984, 1999, 2000 and 2001), of which three had bulbar onset and two had spinal onset. One of these five individuals was still playing soccer when the symptoms began, while for the other four, there was a period of between 4 and 19 years between the end of their professional activities and the onset of ALS. By December 31, 2003, four of these individuals had died (with a mean disease duration of 32.8 months for these four cases) and one individual was alive. ALS may have occurred in these players for a variety of reasons: excessive physical exercise; correlation with trauma or microtrauma; use of illegal toxins for improving athletic performance; or exposure to fertilizers and herbicides use on soccer fields.^28^

### Respiratory failure

Among the studies on treatments related to alterations in the respiratory system, Miller et al.^29^ demonstrated in their study on patients with ALS that early diagnosis of respiratory muscle failure is not possible using the signs and symptoms of the disease. This makes it difficult to decide on the appropriate time for starting to use ventilatory support. Knowing the great dependence that patients on mechanical ventilation acquire, the medical team should explain the advantages and disadvantages of using this support, to patients and caregivers, thus making patients responsible for starting the process.

The types of ventilatory assistance utilized are: non-invasive (continuous positive airway pressure, CPAP; and bilevel positive airway pressure, BiPAP^®^) and invasive mechanical ventilation. Non-invasive assistance uses a nasal or facial mask without the need for endotracheal intubation and has the advantage of avoiding respiratory insufficiency in some cases. In the BiPAP^®^ system, the inspiration and expiration parameters can be controlled, while in CPAP there is only continuous respiratory flow. Invasive ventilation is provided through orotracheal or endotracheal tubes when there is respiratory insufficiency. The aim is to optimize gas exchange and reduce respiratory work; however, greater care is required, which makes this resource expensive, and makes weaning off difficult.^29^

Kleopa et al.^30^ analyzed 52 men and 70 women with a mean age of 62.2 years, between 1993 and 1997, who were divided into three groups regarding respiratory capacity in advanced stages of ALS, according to BiPAP^®^ use. They compared the three groups and observed the differences in survival. The patients in Group 1 tolerated a BiPAP^®^ ventilator for four hours daily and obtained mean survival of 14.2 months beyond the start of the study; the patients in Group 2 used a BiPAP^®^ ventilator for less than four hours daily and obtained seven months of survival; and the patients in Group 3 did not utilize the apparatus and had mean survival of 4.6 months. These authors concluded that patients in the advanced phase of ALS with respiratory discomfort and forced vital capacity of less than 50% may increase their survival time and have better quality of life using BiPAP^®^ ventilators.

Brooks^31^ evaluated 14 ALS patients under 75 years old between 1994 and 1996, with all the patients evaluated using the Spinal Scale (SNS) and Norris Bulbar Scale (BNS)^31^ at three-month intervals over one year. The patients were divided into two groups based on previous arterial blood gases and spirometry results: Group I composed of five women and two men with a mean age of 57.7 years and Group II with three women and four men with a mean age of 62.3 years. These authors concluded that patients with ALS presented forced vital capacity and vital capacity that was at least 50% lower than in healthy individuals, and that those with respiratory insufficiency presented sleep-related disorders such as nocturnal de-saturation and reduced rapid eye movement (REM) phases of sleep. They recommended the use of noninvasive ventilation (BiPAP^®^), as ventilatory support for increasing oxygenation, but they also warned that patients with bulbar-type ALS do not achieve good results with BiPAP^®^ ventilation because of the excessive accumulation of secretion in the advanced stage of the disease.^32^

Bourke et al.^33^ found that the presence of respiratory musculature failure, hypoventilation and sleep disorders in patients with ALS provoked chronic morning headaches, diurnal somnolence, lethargy, fatigue, difficulties in concentration and lack of appetite. Death in these cases was generally by respiratory failure, and noninvasive mechanical ventilation possibly increased survival, although the impact on the quality of life was not very clear and the optimization criteria for starting the treatment were uncertain.

CPAP, the other noninvasive mechanical ventilation resource utilized in patients with ALS, was studied by Barthlen et al.^34^ in two male patients who underwent polysomnography because they presented inefficient sleep quality. The first, a 61-year-old, presented predisposition to muscle fatigue and nocturnal desaturation of up to 62%. The second, a 56year-old, had weakness and muscle fatigue, dysphonia and a range of saturation from 83% to 86%. The CPAP parameters were progressive, according to the patients’ needs. For the first patient, the pressures were between 4 cm H_2_0 and 12 cm H_2_0, over a period of 292 minutes, using a facial mask. The results showed an improvement in nocturnal ventilation, saturation and quality of sleep. The second patient underwent CPAP for one hour at a pressure of 3 cm H_2_O. He was intolerant to the treatment, with weakness of the facial musculature, and was unable to adapt to the mask types. These authors suggested that noninvasive mechanical ventilation could be used to improve the quality of patients’ sleep and respiratory function, independent of whether it was CPAP or BiPAP^®^.

González-Lorenzo and Díaz-Lobato^6^ discussed the indication of the use of invasive mechanical ventilation in medical and family settings. They also discussed questions concerning the benefits of this type of support, with the aim of letting patients decide on their manner of treatment, with full awareness and authorization regarding whether or not their suffering should be prolonged by using this ventilation support. However, discontinuation of support conflicts with questions of medical ethics and social differences.

In the article by Dal Bello-Haas et al.,^7^ functional mobility was assessed over the course of disease evolution, in relation to the benefit of allowing patients to be independent and in accordance with the respective stages of the disease, to facilitate follow-up guidelines for physiotherapists and the multidisciplinary team. It is believed that functional mobility may also be evaluated by predefined functions observed during day-today activities. The proposal in their study, for verifying the evolution of the disease together with directed follow-up, is currently used in large multidisciplinary centers and this policy should be maintained.

Gómez Fernández and Calzada Sierra,^14^ Piemonte and Ramirez^15^ and Duran et al.^17^ were concerned with maintaining good functional levels for as long as possible and preventing complications due to muscle inactivity, through a multidisciplinary approach. These authors evaluated the stages of the disease and proposed selected exercises for each of them, with emphasis on training and guidance for family members and caregivers, and on cost reduction. This is an interesting approach accepted by large centers with multidisciplinary research.

In the study by Pedroso et al.,^16^ it was observed that preventative work reduced the harm to muscle strength, fatigue and functionality. A greater number of patients under treatment for a longer evaluation period would be needed, in order to provide more reliable conclusions.

The study by Drory et al.^18^ was considered inconclusive because of the short time for which the exercise program was applied to the patients, and the small number of patients who continued the exercises to the end of the study period. On the other hand, there seemed to be no worsening of fatigue among the patients who exercised, which was a positive point from the data presented.

## FINAL CONSIDERATIONS

Studies that have reported a relationship between ALS and strenuous exercise among athletes have stimulated researchers to study this theme further, but this hypothesis cannot be confirmed because of the low statistical significance in the few works published. Further studies are required with regard to fatigue and physical exhaustion among people who are not affected by this disease. Moderate and regular exercise monitored by professionals and trained caregivers is considered important for patients with ALS.

Prevention of the probable symptoms inherent to the evolution of the disease at an early stage is a challenge for multidisciplinary teams. Likewise, patients and caregivers must not be left disheartened when faced with difficulties.

Motor and respiratory kinesiotherapy act to improve patients’ quality of life and maintain their functionality for a longer time, and especially when physiotherapists encourage patients and caregivers to perform the recommended exercises at home too.

There have been few studies on the benefits of and indications for the different types of mechanical ventilation for patients with ALS. Invasive ventilation has the aim of extending patients’ survival, but it is restrictive because of the difficulties in weaning off this type of ventilation. On the other hand, the use of BiPAP^®^ and CPAP is indicated for respiratory insufficiency, and these provide improvement in hypoventilation and nocturnal desaturation. However, these techniques depend on individual patients’ tolerance, and studies to prove the efficacy of the apparatus are necessary.

Multidisciplinary teams are able to suggest control measures regarding the evolution of the disease, through the monthly use of scales that assess patients’ functionality and quality of life, in order to improve interventions. With regard to study centers, there is much ongoing research on stem cells from umbilical cord blood, together with research using pharmacological treatments, both in animal models and *in vitro* experiments. Nonetheless, there are further obstacles to overcome, such as the lack of resources in hospitals, insufficient money to perform research and the absence of financial incentives, in the search for concrete results and efficient treatment, or even a cure for amyotrophic lateral sclerosis.

## CONCLUSION

There are few current experimental studies within the field of physiotherapy aimed at finding treatment methods and analyzing these new methods, and particularly with regard to longitudinal studies with adequate numbers of patients. Thus, further studies by physiotherapists are necessary.
